# Retrograde endovascular revascularization for chronic total occlusion of the internal carotid artery: a case report

**DOI:** 10.1007/s00701-021-04875-3

**Published:** 2021-05-20

**Authors:** Takeshi Uno, Masaaki Shojima, Yuta Oyama, Fumitaka Yamane, Akira Matsuno

**Affiliations:** 1 Department of Neurosurgery, Teikyo University School of Medicine, 2-11-1 Kaga, Itabashi-ku, Tokyo, Japan; 2 Department of Neurosurgery, Saitama Medical Center, Saitama Medical University, 1981 Kamoda, Kawagoe-shi, Saitama, Japan

**Keywords:** Endovascular revascularization, Retrograde approach, ICA occlusion, Chronic total occlusion, Transient ischemic attacks

## Abstract

Endovascular revascularization of a chronically occluded internal carotid artery (ICA) is challenging because the occlusive segment can be long and tortuous. A case is presented of a successful recanalization of a chronically occluded ICA by retrograde passing of a guidewire from the intracranial ICA to the cervical ICA via the posterior communicating artery. This case suggests that a retrograde approach for reopening an occluded artery may be useful during neurovascular interventions, similar to percutaneous coronary interventions. In this patient, daily transient ischemic attacks disappeared after successful recanalization of the ICA.

## Introduction

Patients with chronic internal carotid artery (ICA) occlusion and severe cerebral hypoperfusion have a 20% chance of cerebral ischemic events per year, despite medical treatment [6]. Endovascular procedures for ICA occlusions are sometimes performed, with successful revascularization leading to increased perfusion and improved clinical symptoms, including preserving cognitive function [3, 12, 16]. Nevertheless, these procedures succeed in only about 60 to 80% of patients [19]. For the percutaneous treatment of chronic coronary artery total occlusions, a retrograde approach is useful when an antegrade approach is difficult [14]. For the first time in the neurovascular field, we report a retrograde revascularization technique to treat a chronic total occlusion of the ICA.

## Clinical presentation

**Fig. 1:**
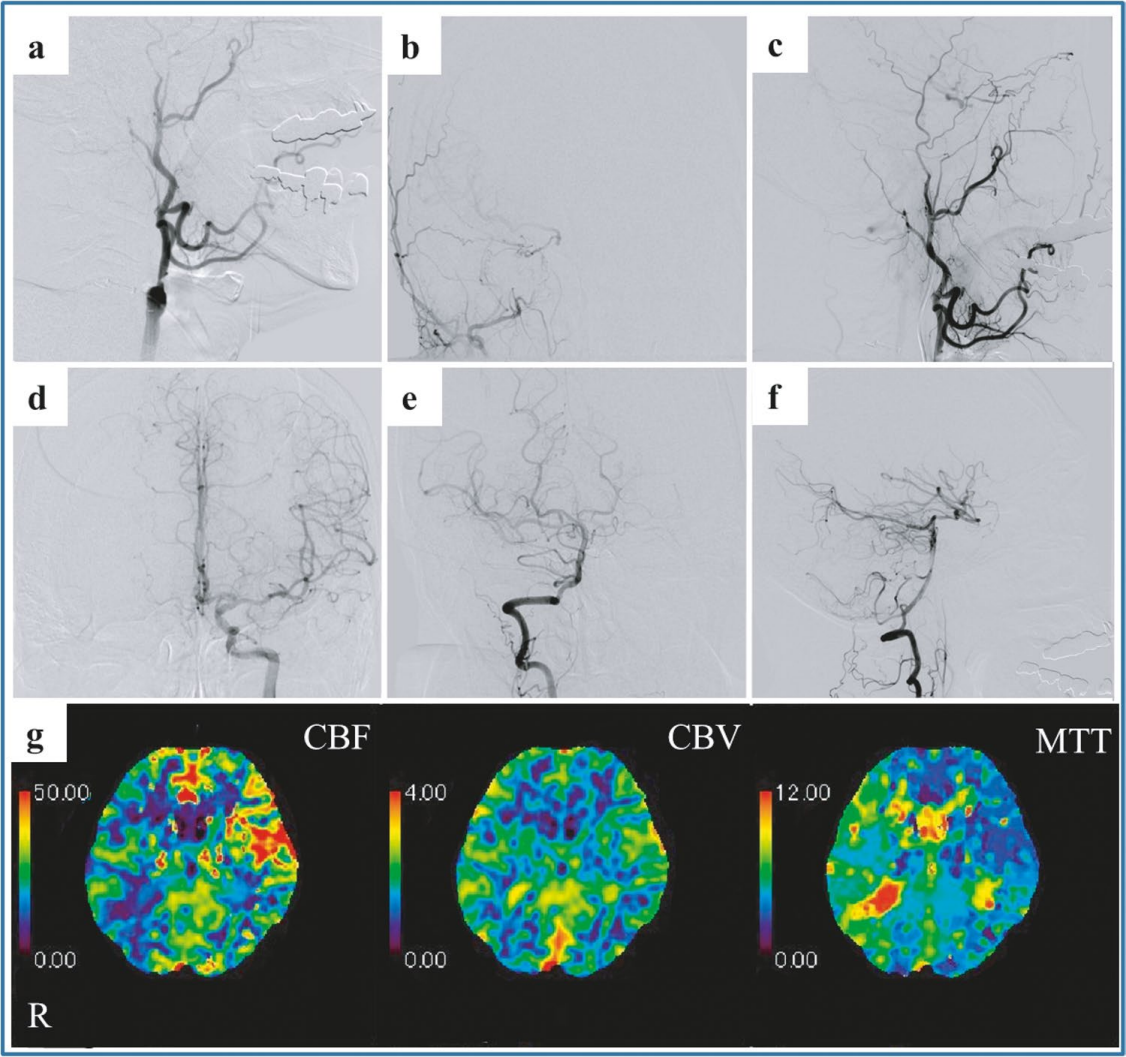
Angiography and perfusion computed tomography before treatment. (**a**–**c**) The right common carotid angiography revealed complete right ICA occlusion and formation of collateral circulation through reversed flow in the right ophthalmic artery from the right external carotid artery. (**d**) The left internal carotid angiography revealed closure of the right A1. (**e**–**f**) The right vertebral angiography revealed the right posterior communicating artery from the posterior circulation. (**g**) Perfusion computed tomography revealed the right cerebral hypoperfusion compared to the left

A 52-year-old man with multiple stenting procedures for arteriosclerosis obliterans in bilateral lower extremities history presented with repeated transient left-sided hemiparesis. Magnetic resonance imaging (MRI) and contrast-enhanced computed tomography imaging detected occlusion of the right ICA and hypoperfusion of the right cerebrum. An endovascular revascularization procedure was recommended owing to daily occurrences of transient ischemic attacks (TIAs) despite treatment with acetylsalicylic acid (100 mg) and cilostazol (200 mg). Cerebral angiography revealed complete occlusion of the right ICA (Fig. 1). The closure of the right A1 and formation of collateral circulation through reversed flow in the right ophthalmic artery from the right external carotid artery and the right posterior communicating artery from the posterior circulation. Although the patient refused EC-IC bypass surgery management, he agreed to receive percutaneous revascularization when endovascular treatment was presented as an alternative.

**Fig. 2:**
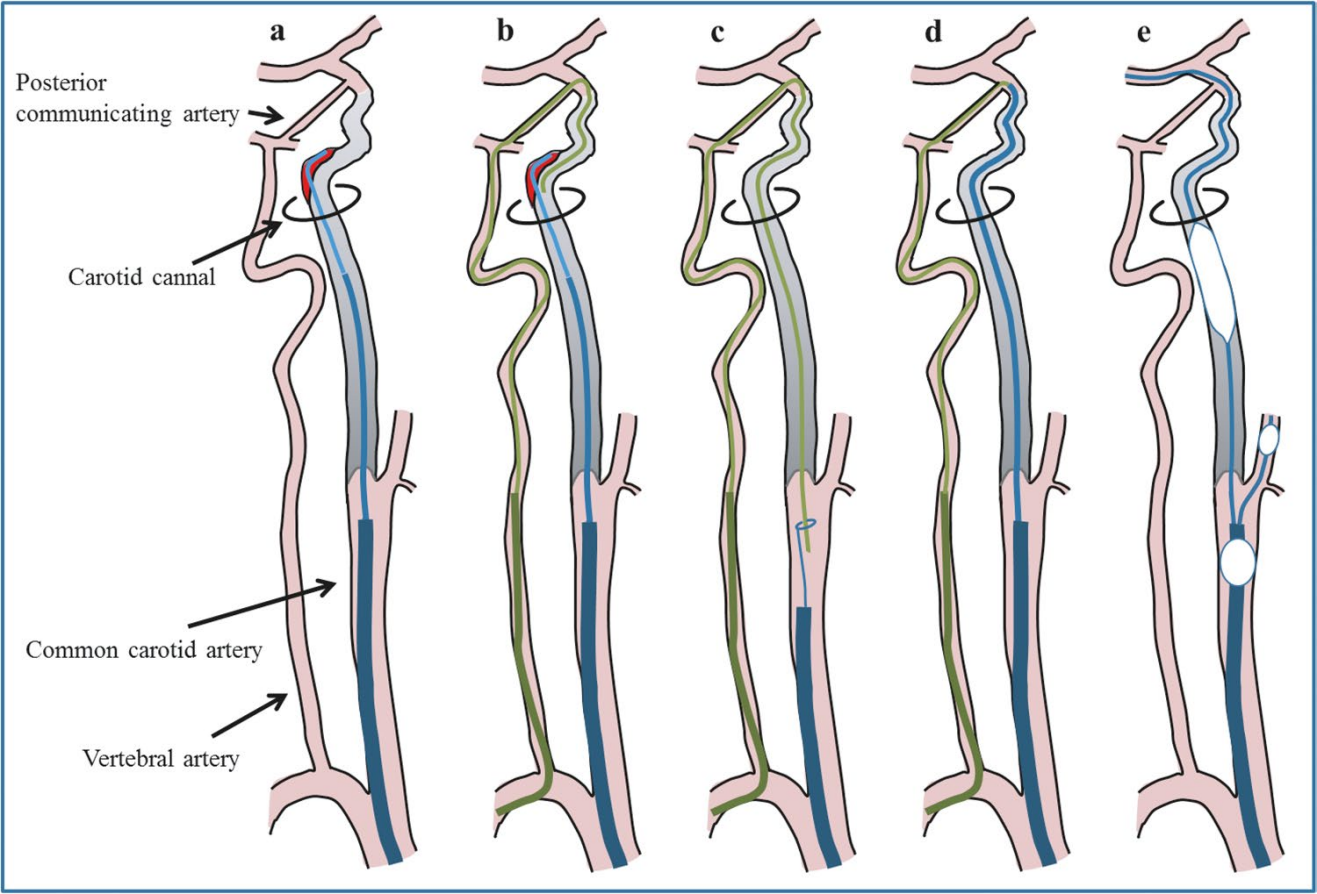
Illustration of the retrograde recanalization procedure. (**a**) In the antegrade procedure, the guidewire broke into the false lumen distal to the carotid canal. (**b**) The guidewire was then retrogradely advanced from the vertebral artery to the internal carotid artery via the posterior communicating artery. The distal fibrous cap was soft, and the guidewire was easily advanced retrogradely. (**c**) The tip of the retrograde guidewire was pulled into the guiding catheter in the common carotid artery with a snare catheter. (**d**) Along the retrograde guidewire, the catheter from the proximal internal carotid artery was passed antegradely across the occluded segment to the distal internal carotid artery. (**e**) The guidewire was navigated antegradely for the middle cerebral artery, and the occluded segment was dilated with the balloon under proximal protection

Under local anesthesia, the revascularization procedure was performed via a percutaneous transfemoral route. First, an antegrade approach was attempted to pass the guidewire through the occlusive lesion. After an 8-F sheath introducer was placed in the right common femoral artery, heparin was maintained intravenously to keep the activated clotting time > 250 s. An 8-F balloon guiding catheter (FlowGate2; Stryker, Tokyo, Japan) was then positioned in the right common carotid artery to prevent distal embolization. Initially, 0.014- and 0.018-in. hydrophilic guidewires (CHIKAI Black; Asahi intecc, Aichi, Japan) were used to attempt to enter the occlusion site with the support of a microcatheter (Excelsior 1018 straight; Boston Scientific, Natick, MA, USA). The fibrous cap at the occluded vessel's proximal part was too hard to penetrate with these guidewires. Therefore, this cap was breached with a stiff 0.018-in. guidewire (Treasure; Asahi intecc, Aichi, Japan). While carefully rotating, the 0.014-in. guidewire was then advanced in the occluded vessel's lumen, followed by the microcatheter. At around the 90° flexion point of the ICA at the entrance to the skull, the guidewire’s smooth progression ceased, with angiography revealing that the catheter had entered the vascular dissection cavity (Fig. 2). Another guidewire was inserted in parallel [9], and the microcatheter was shaped to change the direction of the wire; however, the true lumen of the blood vessel could not be secured.

An antegrade approach was then considered inappropriate. Instead, a retrograde approach was attempted from the direction of the catheter access point from the distal to the proximal ICA (Fig. 2). A 6-F sheathless guiding catheter (Medikit, Tokyo, Japan) was placed in the right vertebral artery via the right radial artery. A 0.014-in. hydrophilic guidewire within a microcatheter was then navigated to the right ICA distal to the occlusion site through the collateral route of the posterior communicating artery. The fibrous cap in the distal part of the occluded vessel was not hard; therefore, the guidewire passed through with only slight manipulation. The guidewire and microcatheter were then easily passed through the obstruction to the cervical ICA, and blood return from the common carotid artery was confirmed through the microcatheter. The 0.014-in. guidewire was then exchanged with a 0.010-in. guidewire of 300-cm length (CHIKAI 10; Asahi intecc, Aichi, Japan). The tip of this guidewire was caught by a 4-mm snare device (Amplatz gooseneck; Medtronic, Minnesota, USA) and pulled through the sheath at the right femoral artery, passing from the radial artery through the occluded vessel to the femoral artery. Subsequently, the microcatheter was placed in the proximal to the distal direction of the ICA to carry devices, such as balloons or stents. A microcatheter (Excelsior 1018 straight; Boston Scientific, Natick, MA, USA) was advanced over the 0.010-in. guidewire into the right distal true ICA through the occluded segment, with great care not to move the intracranial vessels. The microcatheter was then passed through the occluded vessel and reached the distal true lumen of the ICA as if it had been guided in an antegrade fashion.

**Fig. 3:**
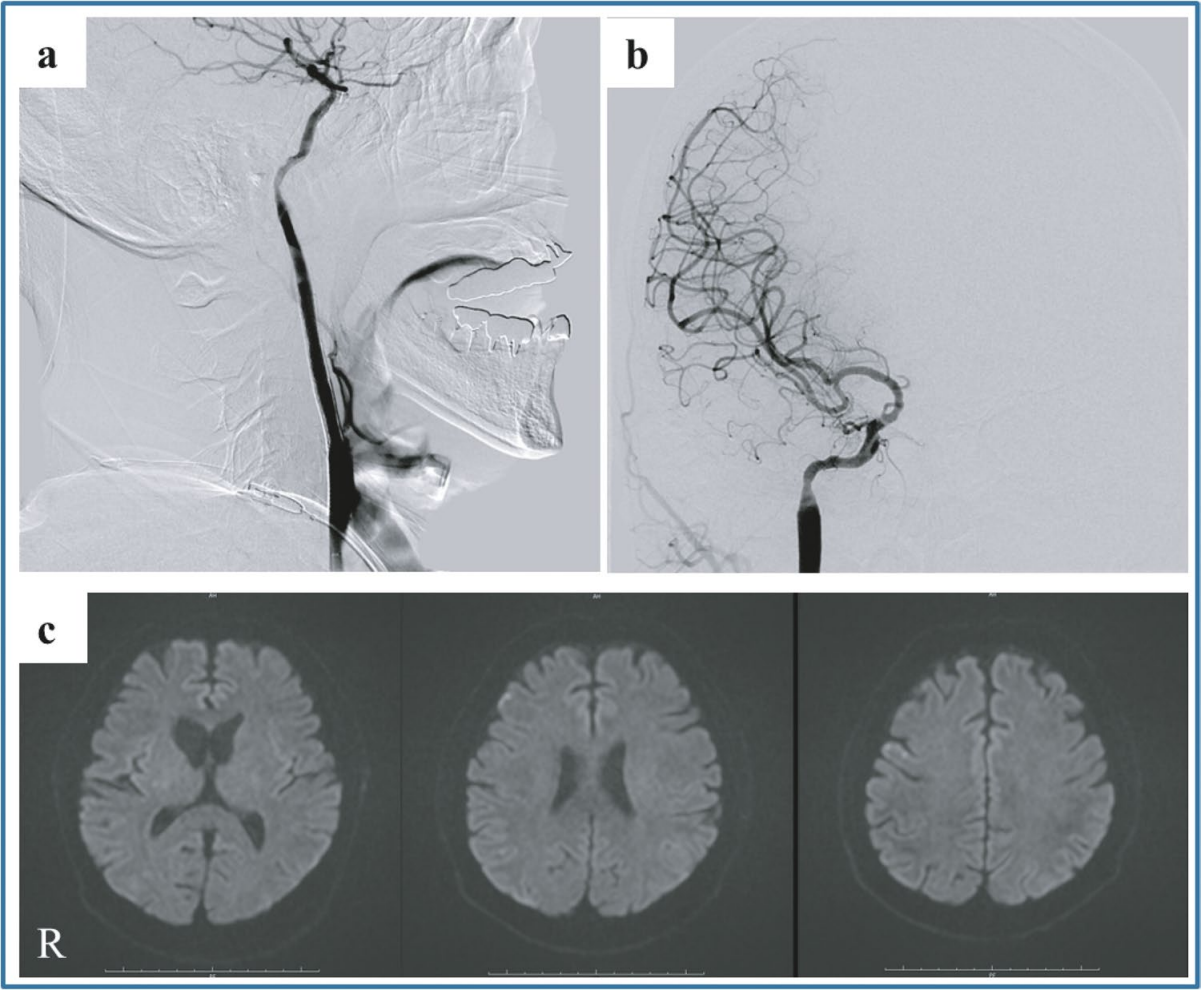
Image after treatment. (**a**–**b**) The right common carotid angiography revealed patency of the right internal carotid artery after 3 months. (**c**) Diffusion-weighted magnetic resonance image 1 day after the procedure revealed small ischemic lesions in the ipsilateral cerebral hemisphere

The subsequent procedure was conducted following the procedure by Shojima et al. [12]. Angiography at the end of the procedure showed antegrade blood flow of the ICA. The patient was discharged 4 days later without neurological deficits. Angiography after 3 months and carotid ultrasonography after 6 months showed antegrade ICA blood flow, without additional TIAs after revascularization (Fig. 3).

## Discussion

This report described a successful retrograde revascularization technique through the posterior communicating artery for chronic ICA total occlusions. Endovascular techniques for treating chronic ICA total occlusions are generally beneficial; however, these procedures can be difficult [5], and generally depend on whether the guidewire can successfully be passed through the occluded segment to enter the vessel's true lumen [1, 5]. If a pseudo-vessel lumen is entered, blood vessel perforation can occur, leading to intracranial hemorrhage and death [19]. Until now, such a procedure would generally have been stopped if the true vessel lumen could not be entered using an antegrade approach. However, a retrograde crossing of a chronically occluded segment could be easier than an antegrade crossing because the distal fibrous cap is generally thinner and softer than the proximal cap [4, 8]. In this case, a stiff 0.018-in. guidewire was required to penetrate the proximal fibrous cap, while the distal fibrous cap was penetrated with only a floppy, hydrophilic 0.014-in. guidewire.

Retrograde procedures are becoming more common during coronary interventions, despite no previous report on ICA occlusion [18]. In these cases, the ‘retrograde’ pathway is generally through a grafted blood vessel from previous bypass surgery or a collateral circulation channel, such as septal or epicardial collateral. Guidewires with good torque performance and microcatheters specialized for retrograde approaches have dramatically increased success rates for coronary interventions [17]. Recanalization of chronic total occlusion in coronary intervention improves angina, enhances athletic performance, and reduces mortality and the need for subsequent coronary artery bypass surgery [7, 11, 13]. The success rate of the combined antegrade and retrograde approach has risen to about 90% in recent years from the 70% success rate with antegrade approach alone [2, 10, 15]. This case suggests that a similar retrograde procedure may be useful for recanalizing chronically occluded ICAs as in the coronary intervention area.

Guiding a carotid stent through an intracranial vessel is not always possible owing to the high risk of vascular rupture. Therefore, we entered the true lumen from the occluded vessel's distal end, pulling the stent from the proximal to the distal end with a snare. Pulling up the catheter is likely to move the blood vessel; thus, great care must be taken during this stage. Furthermore, bypass surgeries can also be performed for cerebral revascularization when appropriate.

## Conclusion

In this case, the patient’s daily TIAs disappeared completely. His course was good, suggesting that a retrograde technique may be useful not only for percutaneous coronary interventions but also for neurovascular interventions when antegrade revascularization procedures are difficult. Thus, the feasibility and safety of this technique should be assessed through larger-scale studies in the future.

## Abbreviations

ICA: Internal carotid artery
TIA: Transient ischemic attacks
MRI: Magnetic resonance imaging
